# Economic burden of multimorbidity among older adults: impact on healthcare and societal costs

**DOI:** 10.1186/s12913-016-1421-7

**Published:** 2016-05-10

**Authors:** Louisa Picco, Evanthia Achilla, Edimansyah Abdin, Siow Ann Chong, Janhavi Ajit Vaingankar, Paul McCrone, Hong Choon Chua, Derrick Heng, Harish Magadi, Li Ling Ng, Martin Prince, Mythily Subramaniam

**Affiliations:** Research Division, Institute of Mental Health, Singapore, Singapore; King’s Health Economics, King’s College London, London, UK; Institute of Mental Health, Singapore, Singapore; Ministry of Health, Singapore, Singapore; Department of Geriatric Psychiatry, Institute of Mental Health, Singapore, Singapore; Changi General Hospital, Singapore, Singapore; Institute of Psychiatry, King’s College London, London, UK

**Keywords:** Multimorbidity, Burden, Healthcare utilisation, Societal cost, Singapore

## Abstract

**Background:**

Multimorbidity is not uncommon and the associated impact it places on healthcare utilisation and societal costs is of increased concern. The aim of the current study was to estimate the economic burden of multimorbidity among older adults in Singapore by investigating its association with the healthcare and societal resource use and cost.

**Methods:**

The Well-being of the Singapore Elderly (WiSE) study was a single phase, cross sectional survey among a nationally representative sample of Singapore residents (*N* = 2565) aged 60 years and above. Multimorbidity was defined in this study as having two or more chronic conditions, from a list of 10 conditions. Care was classified into healthcare which included direct medical care, intermediate and long-term care, indirect care, and social care, provided by paid caregivers and family members or friends. Costs were calculated from the societal perspective, including healthcare and social care costs, by multiplying each service unit with the relevant unit cost. Generalized linear models were used to investigate the relationship between total annual costs and various socio-demographic factors.

**Results:**

The prevalence of multimorbidity was 51.5 %. Multimorbid respondents utilised more healthcare and social care resources than those with one or no chronic conditions. The total societal cost of multimorbidity equated to SGD$15,148 per person, annually, while for those with one or no chronic conditions the total annual societal costs per person were SGD$5,610 and SGD$2,806, respectively. Each additional chronic condition was associated with increased healthcare (SGD$2,265) and social care costs (SGD$3,177). Older age (i.e. 75–84 years old, and especially over 85 years), Indian ethnicity and being retired were significantly associated with higher total costs from the societal perspective, while older age (75 years and above) and ‘Other’ ethnicity were significantly associated with higher total healthcare costs.

**Conclusion:**

Multimorbidity was associated with substantially higher healthcare utilisation and social care costs among older adults in Singapore. With the prevalence of multimorbidity increasing, especially as the population ages, we need healthcare systems that are evolving to address the emerging challenges associated with multimorbidity and the respective healthcare and societal costs.

## Background

Currently healthcare systems are designed around a single-disease framework, however as our population ages, we are seeing a steady increase in the number of people who have multiple co-occurring chronic conditions. People with multiple chronic conditions or multimorbidity have different clinical needs from people who just have one chronic condition and this poses problems when we have a healthcare system that is focused on diseases rather than individuals. For people with multiple conditions, there is often duplication, in a system which is inefficient, burdensome and overwhelming, as a consequence of poor coordination and integration [1, 2].

To date, there is little consensus as to how multimorbidity is defined, however most commonly it refers to the coexistence of two or more chronic conditions in the same individual [3–7]. Multimorbidity prevalence data tends to vary greatly across the world [8] as does the methodology of data collection [1, 2, 9, 10]. The majority of studies focus on a small number of morbidities, and either an older population or hospital population. There is also a lack of clear and comprehensive criteria for the selection of chronic conditions and there is no agreement on the number or type of diseases to be included [8], making comparisons across studies difficult.

Despite these inconsistencies in methodology, terminology and assessment of multimorbidity, the literature has repeatedly shown that multimorbidity is highly prevalent among older adults, with studies frequently reporting prevalence figures between 50–80 % [3, 11, 12]. Multimorbidity is progressively more common with age [5, 13] and is associated with socio-demographic correlates such as gender and socio-economic status [10, 14] as well as reduced functional status [15], poorer quality of life [16] and higher risk of care dependency [17].

More recently there has been an interest in multimorbidity and healthcare utilisation and the associated costs, however the data are still scarce. Furthermore, to our knowledge there has only been a couple of studies that have examined the impact of multimorbidity on healthcare costs from the societal perspective [18, 19], and therefore our study aims to fill this current gap. The extant literature concludes that multimorbidity is associated with increased healthcare utilisation and costs [7] and this burden increases as the number of co-occurring chronic conditions increases [3, 7]. More specifically, multimorbidity has been associated with greater physician visits, hospital visits,  hospitalisations and prescription medications [3, 5, 7, 20]. Multimorbidity is also associated with higher need for specialised care and higher referrals to specialised care [2, 7, 20]. Understanding more about the impact multimorbididty has on healthcare utilisation and associated costs will also be useful for informing future healthcare policies [21].

Singapore is a multi-ethnic, developed country in South East Asia, with a resident population of 3.85 million people, consisting predominantly of Chinese (74.2 %), Malays (13.3 %) and Indians (9.1 %) [22]. The healthcare delivery system in Singapore is dual based, comprising public and private sectors, where affordability of healthcare is ensured with the 3Ms: Medisave, a national mandatory healthcare saving plan; Medishield Life, a national low cost medical insurance scheme for catastrophic illness; and Medifund, where subsidies are provided for needy Singaporeans through a national fund. There is also a special financing system scheme for the elderly population, Eldershield, which provides basic financial protection to those who need long-term care. The healthcare philosophy places a significant emphasis on individual responsibility and the need for co-payment for services provided.

General Practitioners provide 80 % of the primary healthcare services, and doctors in government polyclinics provide the remaining 20 %. Public hospitals (known as restructured hospitals) provide about 80 % of the tertiary care in Singapore, while the remaining care is provided by private hospitals [23]. Patients seeking treatment in public hospitals may apply for a range of subsidies on their total bill, which can be up to 80 % of the total bill; the extent of subsidy received is subjected to guidelines set by the government to allocate limited resources to those who need them most. Subsidies of up to 50 % are also available for specialist outpatient clinics and at polyclinics and up to 75 % for intermediate and long-term care services such as nursing homes [24].

In a previous study, which examined multiple chronic medical conditions among the adult population in Singapore, Subramaniam and colleagues [25], found that 16.3 % of the population had more than one chronic condition. This study however did not examine healthcare utilisation or the associated costs of having multiple chronic conditions and the WiSE study is the first study of its kind in Singapore to explore these associations. The aim of the current study was to estimate the economic burden of multimorbidity among older adults in Singapore by investigating its association with healthcare and societal resource use and cost.

## Methods

### Study design and population

Data for this study were extracted from the Well-being of the Singapore Elderly (WiSE) study, a population-based, cross sectional study which established the prevalence of dementia among residents aged 60 years and above in Singapore. The WiSE study was a single phase, cross sectional survey of Singapore residents (citizens and permanent residents) aged 60 years and above that was conducted between August 2012 and December 2013. Ethical approval was obtained from the relevant institutional review boards (National Healthcare Group Domain Specific Review Board and the SingHealth Centralised Institutional Review Board).

Respondents were randomly selected via a national registry that maintains the names and socio-demographic details such as age, gender, ethnicity and addresses of all residents in Singapore. The sample was inclusive of those residents residing in nursing homes or hospitals at the time of the survey. Residents living outside of Singapore or who were unable to be contacted due to incomplete or incorrect addresses were excluded from the survey.

In addition to each selected respondent, an informant was chosen to also participate in the study. An informant was defined as ‘the person who knows the older person best’ and the actual amount of time spent with the older person was used as a criterion for deciding the best informant in the case where multiple people met this criteria. Both the older person and the informant were administered the respective questionnaires.

Administration of the questionnaires was either in English, Chinese, Malay, Tamil or in one of the Chinese dialects: Cantonese, Hokkien or Teochew. The language used was based on the preference of the person being interviewed. The interview itself took on average two to three hours to complete. Written informed consent was obtained from all respondents and for those older persons who were not cognitively capable of providing informed consent, written consent was obtained from a legally acceptable representative or next of kin. Information pertaining to the WiSE methodology has been described in greater detail in a previous article [26].

### Measures

The respondent was asked a series of questions relating to a number of chronic conditions they have as well as their healthcare utilisation during the three months prior to their interview. The informant version of these questions was administered instead of, or in addition to the respondent version, when the older person was unable to answer the questions reliably.

#### Chronic conditions and multimorbidity

As part of the background socio-demographic and risk factor questionnaire, a chronic conditions checklist was read to respondents and they were asked whether they had any of the following chronic conditions: High blood pressure; Heart trouble (including heart attack, angina, heart failure and valve disease); Stroke; Transient Ischemic Attacks (TIAs); Diabetes; Depression; Arthritis or Rheumatism; Chronic obstructive pulmonary disease (COPD); Breathlessness or asthma; and Cancer. Multimorbidity was defined as two or more of these chronic conditions being present in the one person at the same time [3–7, 20].

#### Healthcare utilisation

Healthcare utilisation data was obtained from the respondents and their informants using an adapted version of the Client Service Receipt Inventory (CSRI) [27], which contains questions about specific community, hospital and informal care services utilised during the three month period prior to the interview. Medical services included community services (e.g. primary care, private doctor, dentistry, and traditional healers), inpatient and outpatient care (e.g. hospitalisations, accident & emergency (A&E) visits), day care centres, respite and nursing home care and medication. Informal care arrangements included time spent with the respondents in assisting them in daily activities, paid care and change in work status due to care provision. Care was classified into; (i) healthcare which includes direct medical care, intermediate and long-term care, indirect care and (ii) social care which was provided by paid caregivers and family members of friends (unpaid caregivers). More details have been reported elsewhere [28].

#### Healthcare costs

Costs were calculated from the societal perspective, including healthcare and social care costs, by multiplying each service unit (i.e. consultations per minute, visits per day) by the service unit cost. To estimate the annual direct medical costs, the amount of money spent on medical services in the past three months, was multiplied by four, assuming that participants utilise resources at the same rate during the year. Due to the scarcity of local unit cost data, an alternative approach was used to estimate the annual healthcare costs. This approach involved the application of UK unit costs for health and social care services and conversion to Singapore dollars, assuming that the relationship between UK and Singapore unit costs is fixed and the ratio of costs between these countries remains unchanged over the years [29].

The process included determination of the reference unit costs (UK) for each specific service [30], generation of ratios for inpatient and outpatient settings between the reference country (UK) and Singapore, using data from the WHO-CHOICE (WHO-CHOosing Interventions that are Cost-Effective) database [31] and application of these ratios to the unit cost of each service in the UK in order to generate country-specific unit costs for Singapore. For other direct medical care including private health care doctors, other private health care workers, dentists, traditional healers, A&E and medication, the average out-of-pocket reported amount of expenses was used instead of applying the ratios to the UK unit cost as they were deemed more representative of the Singapore population. The human capital approach was used as the primary method of valuing unpaid (informal) care. Under this method, the average national wages in Singapore were used as a proxy for valuing care provided by family members or friends. For paid care, due to lack of information on the amount of money paid for home care from the survey, the average per hour wage of a semi-skilled worker was used as unit cost. It should be noted that the cost calculations were based on self-reported service utilisation, which may have under or overestimated the service use and the corresponding costs. For example, it was difficult to account for the amount of subsidized costs, as the variables associated with health insurance premiums were largely incomplete. Therefore, the calculations were based on the reported out-of-pocket amount for some services (e.g. dentist, traditional healer) and on costs assumed to be covered by the national health system (e.g. restructured hospital doctor) for other services. A more detailed description of the methodology used to derive the unit costs has been published elsewhere [28].

### Statistical analysis

Statistical analyses were carried out using the SAS software version 9.2 (SAS Institute Inc., Cary, NC, USA) and STATA version 13.0. To ensure that the survey findings were representative of the older adult population in Singapore, all estimates were analyzed using survey weights to adjust for oversampling, non-response and poststratification according to age and ethnicity of the Singapore population (aged 60 years and above) for the year 2013 [22]. Missing data in categorical and continuous variables were imputed using the last value carried forward and mean imputation methods, respectively. More sophisticated imputation techniques, such as multiple imputations, were not used as the data were mostly complete. Mean and standard errors were calculated for continuous variables, and frequencies and percentages for categorical variables. At a first stage, the annual total healthcare and societal costs were regressed on the multimorbidity groups without controlling for covariates. Similarly, the total costs per additional chronic condition were regressed on the total number of morbidities, without covariate adjustment. At a second stage, the impact of multimorbidity on total healthcare and societal costs was adjusted for socio-demographic variables including age, gender, ethnicity, marital status, education and employment status. Similarly, the calculation of the total costs per additional chronic condition was adjusted for the same covariates. Due to the skewed distribution of costs, the non-normality and heteroskedasticity of residuals, generalized linear models with gamma distribution and log link function were used. The log link function means that the value of the exponentiated coefficients is interpreted as proportional changes of the total societal costs per additional chronic condition. The methodology was similar to that reported by Abdin and colleagues [28]. All statistically significant differences were evaluated at the *p* < 0.05 level using 2-sided t tests.

## Results

A total of 2565 respondents completed the study giving a response rate of 65.6 %. The socio-demographic characteristics of the respondents are shown in Table 1. The sample comprised 55.9 % females and 44.1 % males. The majority of the sample was aged between 60-74 years (75.0 %), of Chinese ethnicity (83.3 %), and currently married (64.0 %). The WiSE study found that the prevalence of multimorbidity was 51.5 %, while the prevalence of only one chronic condition was 29.3 %. A breakdown of prevalence estimates by demographic variables is presented in Table 2. The odds of multimorbidity were significantly higher among those aged 75-84 years, of Indian and ‘Other’ ethnicities, with secondary education and retired, while the odds of one chronic condition were significantly higher among those unemployed (Table 3).

**Table 1:** Socio-demographic characteristics of the sample

Demographic characteristic	Category	*N*	Un-weighted %	Weighted %	SE
Age group	60–74 years	1494	58.2	75	0
	75–84 years	669	26.1	19.5	0
	85+ years	402	15.7	5.5	0
Gender	Men	1117	43.5	44.1	1.4
	Women	1448	56.5	55.9	1.4
Ethnicity	Chinese	1012	39.5	83.3	0
	Malay	745	29	9.3	0
	Indian	772	30.1	6	0
	Others	36	1.4	1.4	0
Marital status	Never married	136	5.3	8	0.8
	Married/cohabiting	1484	57.9	64	1.3
	Widowed	836	32.6	22.5	1
	Divorced/separated	107	4.2	5.5	0.7
Education	None	511	20	16.5	1
	Some, but did not complete primary	620	24.3	23.9	1.2
	Completed primary	640	25.1	24.8	1.2
	Completed secondary	517	20.3	22.4	1.2
	Completed tertiary	262	10.3	12.4	1
Employment	Paid work (part-time and full-time)	688	27.2	33.9	1.3
	Unemployed (looking for work)	32	1.3	1.5	0.4
	Homemaker	808	31.9	26.3	1.2
	Retired	1006	39.7	38.3	1.3

**Table 2:** Prevalence of multimorbidity by socio-demographic characteristics

Variables	2 or more chronic conditions			1 chronic condition			No chronic conditions		
	*n*	%	SE	*n*	%	SE	*n*	%	SE
Overall	1420	51.5	1.4	692	29.3	1.3	453	19.3	1.1
Age group									
60–74 years	770	47.2	1.7	419	31.1	1.6	305	21.6	1.4
75–84 years	419	65.3	2.5	157	23.1	2.2	93	11.6	1.6
85+ years	231	59.8	3.2	116	25.9	2.9	55	14.3	2.3
Gender									
Men	587	49.6	2.1	309	31.2	2	221	19.1	1.7
Women	833	52.9	1.9	383	27.7	1.7	232	19.4	1.6
Ethnicity									
Chinese	540	50.7	1.7	291	29.9	1.5	181	19.4	1.3
Malay	364	48.8	2	220	28.7	1.8	161	22.5	1.7
Indian	491	62.7	1.8	172	21.8	1.6	109	15.5	1.4
Others	25	67.7	7.9	9	26.4	7.2	2	5.9	4.1
Marital status									
Never married	59	41.8	5.3	44	33.5	5.2	33	24.6	4.7
Married/cohabiting	798	50.4	1.8	398	29.4	1.7	288	20.2	1.5
Widowed	500	58.5	2.6	227	28.1	2.4	109	13.4	1.9
Divorced/separated	61	46.7	6.4	23	27.7	5.9	23	25.6	5.7
Education									
None	297	59.2	3.2	136	27.5	3	78	13.4	2.2
Some, but did not complete primary	351	54.6	2.9	165	27.3	2.6	104	18.1	2.3
Completed primary	359	52.7	2.9	164	26.3	2.5	117	21	2.4
Completed secondary	267	44.4	3	148	33.2	2.9	102	22.5	2.6
Completed tertiary	136	44.9	4.2	76	35	4.1	50	20.1	3.4
Employment status									
Paid work (part-time and full-time)	295	41	2.5	205	32.8	2.4	188	26.2	2.3
Unemployed (looking for work)	16	45.8	12	11	46.4	12.1	5	7.8	4.6
Homemaker	485	54	2.7	212	27.8	2.4	111	18.1	2.2
Retired	607	59.3	2.2	255	26.1	2	144	14.5	1.6

**Table 3:** Socio-demographic correlates of multimorbidity

Demographic characteristic	Category	2 or more chronic conditions				1 chronic condition			
		OR^a^	95 % CI		*p* value	OR^a^	95 % CI		*p* value
Age group	60–74 years	Ref							
	75–84 years	1.8	1.2	2.8	0.006	1.2	0.7	1.9	0.559
	85+ years	0.9	0.6	1.7	0.987	0.9	0.5	1.6	0.613
Gender	Men	Ref.				Ref.			
	Women	0.9	0.6	1.3	0.44	0.8	0.5	1.2	0.201
Ethnicity	Chinese	Ref.				Ref.			
	Malay	0.8	0.6	1	0.089	0.8	0.6	1.1	0.141
	Indian	1.7	1.2	2.2	0.001	0.9	0.6	1.3	0.552
	Others	5.1	1	25.1	0.043	3	0.6	14.3	0.176
Marital	Married	Ref.				Ref.			
	Divorced/separated	0.8	0.4	1.6	0.597	0.7	0.3	1.6	0.422
	Never married	0.7	0.4	1.4	0.342	0.9	0.5	1.7	0.778
	Widowed	1.3	0.8	2	0.299	1.5	0.9	2.4	0.139
Education	None	Ref.				Ref.			
	Some, but did not complete primary	0.8	0.5	1.3	0.342	0.8	0.4	1.4	0.352
	Completed primary	0.7	0.4	1.2	0.156	0.6	0.4	1.1	0.123
	Completed secondary	0.6	0.3	0.9	0.047	0.8	0.4	1.4	0.395
	Completed tertiary	0.6	0.3	1.2	0.123	0.9	0.4	1.7	0.659
Employment	Paid work (part-time and full-time)	Ref.				Ref.			
	Homemaker	1.5	0.9	2.5	0.113	1.2	0.7	2.1	0.462
	Retired	2.1	1.4	3.2	0.002	1.3	0.9	2	0.217
	Unemployed (looking for work)	3.5	0.8	15.1	0.087	4.8	1.2	19.9	0.031

^a^Odds ratio (OR) was derived from multinomial logistic regression analysis

The resource utilisation and costs by multimorbidity group are shown in Table 4. Multimorbid respondents utilised more healthcare and social care resources than those with one or no chronic conditions. The multimorbid group had an average of three contacts with polyclinic doctors per year, versus 2.3 and 1.1 for those with only one and no chronic conditions, respectively, while contacts with restructured hospital doctors were also higher among those with multimorbidity (3.2 vs. 1.3 and 1.6 respectively). The mean number of hospital days was also higher for the multimorbid group (2.7 vs. 0.5 and 0.1 days, respectively). It is notable that social care utilisation was substantially higher for the multimorbid group compared with those with one or no chronic conditions, with the hours spent in providing supervision being the major component of high utilisation (98.6 hours vs 14.6 hours and 10.5 hours, respectively).

**Table 4:** Number of service users, annual number of contacts and costs per person for all individuals among everyone in the sample and by multimorbidity group

	Service users	Total study sample		2 or more chronic conditions		1 chronic condition		No chronic conditions	
Healthcare services	*n* (%)	Contacts	Costs	Contacts	Costs	Contacts	Costs	Contacts	Costs
Direct Medical Care									
Primary care (polyclinic doctor)	1,082 (42 %)	2.4 (0.2)	S$231 (19)	3.0 (0.2)	S$303 (29)	2.3 (0.5)	S$202 (42)	1.1 (0.5)	S$81 (12)
Restructured hospital doctor	749 (29 %)	2.3 (0.2)	S$692 (55)	3.2 (0.3)	S$1019 (96)	1.3 (0.2)	S$357 (57)	1.6 (0.6)	S$329 (86)
Other restructured hospital health worker	121 (40 %)	0.5 (0.5)	S$31 (7)	0.7 (0.2)	S$47 (13)	0.3 (0.1)	S$19 (7)	0.1 (0.1)	S$5 (3)
Private hospital/clinic doctor	655 (27 %)	1.7 (0.1)	S$208 (66)	1.9 (0.1)	S$254 (108)	1.8 (0.2)	S$224 (121)	1.1 (0.2)	S$63 (13)
Other private health worker	29 (2 %)	0.3 (0)	S$72 (38)	0.5 (0.2)	S$60 (26)	0.2 (0.1)	S$123 (121)	0.2 (0.1)	S$29 (18)
Dentist	215 (12 %)	0.7 (0.1)	S$369 (151)	0.8 (0.1)	S$146 (36)	0.7 (0.1)	S$940 (511)	0.5 (0.1)	S$101 (45)
Traditional healer	144 (10 %)	1.8 (0.3)	S$59 (12)	2.4 (0.5)	S$75 (21)	1.3 (0.3)	S$49 (15)	0.8 (0.2)	S$33 (11)
Hospital days	156 (4 %)	1.6 (0.3)	S$1,692 (339)	2.7 (0.6)	S$2,920 (638)	0.5 (0.2)	S$587 (263)	0.1 (0)	S$93 (46)
A&E	135 (4 %)	n/a	S$128 (40)	n/a	S$153 (46)	n/a	S$152 (109)	n/a	S$23 (13)
Medication	2,109 (81 %)	n/a	S$478 (47)	n/a	S$675 (89)	n/a	S$345 (34)	n/a	S$153 (19)
Intermediate and Long-Term Care									
Day care centre	19 (1 %)	1.1 (0.4)	S$49 (16)	1.7 (0.6)	S$75 (29)	0.9 (0.6)	S$34 (22)	0	0
Respite care, Nursing home	24 (1 %)	1.8 (0.5)	S$381 (107)	2.2 (0.8)	S$484 (169)	1.4 (0.9)	S$298 (188)	1.1 (0.7)	S$231 (155)
Indirect Medical Care									
Travelling	104 (0 %)	n/a	S$94 (10)	n/a	S$131 (18)	n/a	S$66 (13)	n/a	S$36 (9)
Accompanying	104 (0 %)	n/a	S$240 (20)	n/a	S$325 (36)	n/a	S$181 (24)	n/a	S$102 (15)
Social Care									
ADL (hours) Informal Care									
Dressing	166 (3 %)	13.7 (1.9)	S$248 (34)	22.1 (3.5)	S$398 (63)	4.1 (1.2)	S$83 (26)	5.8 (2.6)	S$101 (44)
Feeding	248 (5 %)	24.7 (2.8)	S$451 (50)	38.3 (5.1)	S$692 (90)	10.1 (2.3)	S$190 (45)	10.8 (3.6)	S$203 (69)
Grooming	130 (2 %)	11.7 (2.1)	S$207 (37)	20.6 (4.1)	S$360 (70)	2.2 (0.9)	S$43 (18)	2.5 (1.1)	S$47 (20)
Toileting	151 (3 %)	15.2 (2.5)	S$275 (44)	25.4 (4.7)	S$462 (83)	3.9 (1.3)	S$75 (26)	4.9 (2.3)	S$82 (39)
Bathing	167 (3 %)	16 (2.4)	S$298 (48)	23.7 (4.4)	S$441 (87)	9 (2.5)	S$165 (45)	6.1 (2.9)	S$114 (58)
IADL (hours) Informal Care									
Communication	292 (6 %)	60.2 (9.1)	S$1111 (164)	94.1 (17)	S$1,744 (308)	28.5 (7.5)	S$505 (128)	18.1 (5)	S$340 (93)
Transportation	94 (2 %)	14.8 (2.7)	S$271 (49)	25.6 (5.2)	S$467 (92)	4.7 (1.6)	S$88 (29)	1.2 (0.7)	S$23 (13)
Supervision	151 (3 %)	57.1 (14.9)	S$1076 (282)	98.6 (28.5)	S$1,856 (540)	14.6 (6.3)	S$266 (112)	10.5 (6.3)	S$222 (141)
Paid Care									
Paid care (during day time)	309 (0 %)	21.2 (1.7)	S$852 (69)	32.6 (3.1)	S$1,312 (125)	11.3 (2.3)	S$455 (94)	5.6 (1.7)	S$227 (67)
Paid care (during night time)	189 (0 %)	11.5 (1.3)	S$464 (53)	18.6 (2.5)	S$747 (99)	4 (1.1)	S$162 (46)	4.2 (1.2)	S$168 (49)

All differences in service use between groups were statistically significant, apart from contacts to private health worker, dentist, and respite care

All differences in costs between groups were statistically significant, apart from costs of private doctor, private health worker, dentist, traditional healer, A&E, and respite care

*ADL* Activities of Daily Living, *IADL* Instrumental Activities of Daily Living

In almost all types of services, multimorbid respondents incurred higher costs than those with one or no chronic conditions. The costs of hospitalisation, contacts with restructured hospital doctors, and medication were the biggest drivers of healthcare costs. With regard to social care; the higher costs were associated with providing supervision and help with communication, and with paid care during the daytime. The cost of these services was three to seven times higher for the multimorbidity group compared with the costs incurred by the two other groups.

Table 5 summarises the total societal cost, according to healthcare and social care costs. The total annual societal cost of multimorbidity equated to SGD$15,148 per person, whereas for those with one or no chronic conditions the total annual societal costs, per person  were SGD$5,610 and SGD$2,806 respectively. The significant increase in total societal cost was more evident among the social care cost which was SGD$8,480 for those with multimorbidity while this was SGD$2,032 and SGD$1,527 for those with one or no chronic conditions, respectively. Table 6 shows the cost per additional chronic condition whereby the total healthcare cost attributed to a SGD$2,265 increase per chronic condition, whilst the social care cost was an additional SGD$3,177 per condition.

**Table 5:** Mean annual costs per person by multimorbidity group

	Total study sample	2 or more chronic conditions	1 chronic condition	No chronic conditions
Healthcare services	Cost (95 % CI)	Cost (95 % CI)	Cost (95 % CI)	Cost (95 % CI)
Direct Medical Care	S$3,962 (3152, 4771)*	S$5,652 (4285, 7019)	S$2,998 (1662, 4334)	S$911 (674, 1147)
Intermediate and Long-Term Care	S$430 (218, 641)*	S$559 (223, 895)	S$333 (−38, 704)	S$231 (−73, 535)
Travelling	S$94 (74, 113)*	S$131 (97, 165)	S$66 (41, 90)	S$36 (19, 53)
Accompanying	S$240 (200, 280)*	S$325 (254, 397)	S$181 (134, 228)	S$102 (73, 131)
Total healthcare cost	S$4,725 (3866, 5584)*	S$6,667 (5220, 8115)	S$3,578 (2165, 4991)	S$1,279 (876, 1683)
ADL	S$1,293 (972, 1614)*	S$2,054 (1456, 2652)	S$495 (269, 721)	S$471 (145, 797)
IADL	S$1,381 (1020, 1743)*	S$2,211 (1531, 2892)	S$593 (326, 859)	S$363 (173, 553)
Unpaid Care	S$3,936 (2842, 5030)*	S$6,421 (4342, 8499)	S$1,415 (830, 2000)	S$1,132 (530, 1734)
Paid Care	S$1,316 (1103, 1530)*	S$2,060 (1663, 2456)	S$617 (388, 845)	S$395 (193, 596)
Total social care cost	S$5,253 (4084, 6421)*	S$8,480 (6263, 10697)	S$2,032 (1345, 2719)	S$1,527 (839, 2214)
Total societal cost	S$9,977 (8339, 11615)*	S$15,148 (12154, 18141)	S$5,610 (3884, 7335)	S$2,806 (1952, 3660)

*ADL* Activities of Daily Living

*IADL* Instrumental Activities of Daily Living

*p-value <0.05

**Table 6:** Total annual costs per person per additional chronic condition

	Unadjusted	Adjusted
Healthcare services	Cost (95 % CI)	Cost (95 % CI)
Direct Medical Care	S$1,744 (1233, 2255)	S$1,317 (879, 1755)
Intermediate and Long-Term Care	S$186 (−36, 407)	S$91 (−127, 309)
Travelling	S$32 (21, 43)	S$38 (21, 55)
Accompanying	S$69 (47, 91)	S$87 (51, 123)
Total healthcare cost	S$2,030 (1450, 2611)	S$2,265 (1354, 3176)
ADL	S$904 (456, 1352)	S$737 (260, 1215)
IADL	S$1,165 (622, 1708)	S$970 (415, 1526)
Unpaid Care	S$3,072 (1739, 4404)	S$2,574 (1283, 3866)
Paid Care	S$878 (638, 1118)	S$602 (401, 803)
Total social care cost	S$3,950 (2519, 5381)	S$3,177 (672, 1826)
Total societal cost	S$5,980 (4310, 7651)	S$6,022 (3624, 8420)

*ADL* Activities of Daily Living

*IADL* Instrumental Activities of Daily Living

The outputs of the generalised linear regressions are shown in Table 7. The results reveal that being older than 75 years, and especially over 85 years, of Indian ethnicity, and retirement were significantly associated with higher total costs from the societal perspective. In particular, being between 75–84 years old would increase costs by approximately 80 %, while being over 85 years old would result in a five-fold increase, from the societal perspective. Indian ethnicity (versus Chinese) was predicted to increase costs by 36 %, while retirement would increase societal costs by more than 100 %. On the other hand, lower societal costs were associated with some (but did not complete primary school) education, with a decrease in costs by 29 %. From the healthcare perspective, age was also significantly associated with increased costs; being aged 75–84 years and 85 years and above would increase healthcare costs by 48 % and 261 % respectively. We found that the adjusted R-squares of the regression model for the healthcare and societal costs were 1.8 % and 7.2 %, respectively.

**Table 7:** Predictors of total costs based on generalised linear regression with gamma distribution

	Societal costs					Healthcare costs				
Independent variables	Exp. coeff	95 % CI		*p*-value	Association with total costs	Exp. coeff	95 % CI		*p*-value	Association with total costs
Total number of morbidities	1.67	1.50	1.86	0.001	67 %	1.53	1.38	1.70	0.001	53 %
Age Group										
60–74 years	Ref.					Ref.				
75–84 years	1.79	1.25	2.58	0.002	79 %	1.48	1.01	2.19	0.046	48 %
85+ years	5.27	3.50	7.91	0.001	427 %	3.61	2.16	6.03	0.001	261 %
Gender										
Male	Ref.					Ref.				
Female	0.97	0.62	1.53	0.902	−3 %	0.98	0.64	1.51	0.933	−2 %
Ethnicity										
Chinese	Ref.					Ref.				
Malay	1.27	0.92	1.76	0.144	27 %	0.94	0.66	1.34	0.729	−6 %
Indian	1.36	1.01	1.84	0.045	36 %	1.20	0.85	1.69	0.310	20 %
Others	3.14	0.99	9.95	0.052	214 %	4.28	1.22	15.01	0.023	328 %
Marital Status										
Never married	Ref.					Ref.				
Married/Cohabiting	0.78	0.32	1.94	0.599	−22 %	1.00	0.36	2.77	0.993	0 %
Widowed	0.82	0.34	1.99	0.656	−18 %	0.83	0.30	2.34	0.727	−17 %
Divorced/Separated	0.58	0.20	1.63	0.300	−42 %	0.64	0.22	1.83	0.406	−36 %
Education										
None	Ref.					Ref.				
Some (did not complete primary)	0.71	0.51	0.99	0.041	−29 %	0.79	0.55	1.15	0.215	−21 %
Completed primary	1.15	0.70	1.89	0.588	15 %	1.34	0.81	2.22	0.261	34 %
Completed secondary	1.60	0.88	2.89	0.121	60 %	1.72	0.99	3.01	0.055	72 %
Completed tertiary	1.19	0.72	1.97	0.495	19 %	1.37	0.80	2.35	0.251	37 %
Employment status										
Paid work (part-time and full-time)	Ref.					Ref.				
Unemployed	0.76	0.28	2.03	0.582	−24 %	0.89	0.30	2.66	0.833	−11 %
Homemaker	1.62	0.89	2.96	0.118	62 %	0.93	0.56	1.53	0.767	−7 %
Retired	2.39	1.44	3.96	0.001	139 %	1.33	0.82	2.18	0.251	33 %
Constant	1,493	583	3,826	0.001	-	1,251	425	3,685	0.001	-
Goodness of fit indices										
Adjusted R square	0.072					0.018				
Akaike Information Criterion (AIC)	4263.57					4040.45				
Bayesian Information Criterion (BIC)	1346065					1124374				

## Discussion

This is the first study in Singapore to provide a comprehensive estimate of both the healthcare and societal cost of multimorbidity among residents aged 60 years and above, via a nationally representative sample. We found the prevalence of multimorbidity to be 51.5 % and this was significantly associated with age (75–84 years versus 60–74 years), ethnicity (Indian and ‘other’ ethnicities versus Chinese) education (secondary education versus no education) and being retired versus those in paid full time or part time work.

According to a recent systematic review [32], multimorbidity prevalence rates among older adults have been reported to be between 55 %–98 %, while in our study the prevalence was 51.5 %. This discrepancy might be due to different age-groups included in different studies, differences in the number of chronic conditions investigated, differences in the study settings as well as different means of assessing the presence of diseases. Increasing age has been consistently associated with multimorbidity, while the increased prevalence of multimorbidity among those who are retired, could be explained by the fact that having multiple chronic conditions has reduced the ability of older adults to work. Multimorbidity was also significantly more prevalent among those of Indian ethnicity and numerous studies have similarly noted ethnic differences [24, 33] which could be a result of underlying cultural, genetic or environmental factors.

With regards to the association between education and multimorbidity, results commonly vary across low, middle and high income countries. In high income countries similar to Singapore, an inverse relationship between multimorbidity and education is commonly seen where multimorbidity is more prevalent among individuals with lower education [34–36]. Whilst we observed a gradient in the odds of multimorbidity across educational levels, not all findings were significant and therefore a more in depth exploration of the impact of education on multimorbidity is needed.

There were considerable variations in healthcare utilisation and health and social care costs among those with and without multimorbidity. Multimorbidity was associated with increased healthcare utilisation, including more contacts with general practitioners, hospital doctors, increased A&E admissions and medications taken, compared to those with just one or no chronic conditions, and this is consistent with previous research [3, 20, 37]. Whilst intermediate and long term care (i.e. day care centres, nursing homes or respite care) and indirect medical care (time spent travelling and accompanying someone) were not frequently used among the total sample, these were utilised mostly by those who were multimorbid. In addition to medical care utilisation, multimorbidity was also associated with increased social care utilisation in terms of help provided with activities of daily living (ADLs) such as dressing, feeding and grooming and instrumental activities of daily living (IADLs) including communication, transport and supervision, when compared to those with one or no chronic conditions.

Overall, we found that the total annual cost per person of multimorbidity was SGD$15,148 and this was driven slightly more by social care costs (SGD$8,480), as opposed to healthcare costs (SGD$6,667). When weighted back to the entire Singapore population aged 60 years and above, the total cost of multimorbidity equates to SGD$ 4.37 billion per year, highlighting the magnitude of the problem and associated burden. There was also a significant increase in the social care cost when comparing those with no, one or two or more comorbidities; the social care cost of having no chronic conditions was SGD$1,527, SGD$2,032 for one chronic condition, whilst this increased drastically to SGD$8,480 for those with multimorbidity. To our knowledge, there are only a couple of published studies examining the impact of multimorbidity on costs from the societal perspective. A German study [18] examined the impact of multimorbidity on healthcare costs including a social care cost component of nursing care among the elderly. A Dutch study among elderly patients [19], examined the societal costs of patients at risk of poor functioning one year after hospital discharge and found that formal and informal care showed a similar trend with increasing risk. The findings of these two European studies could not be directly compared with our study findings as both studies employed different methods to measure, quantify and define informal care. However, it is notable that the contribution of informal care to the total costs is quite similar to that of the healthcare costs, or slightly increased in specific cases. This highlights the importance of informal care such as that provided by family members or carers in the community. In order to avoid caregiver burnout and stress, future interventions need to not only be patient-centred and address the needs of those with multimorbidity but also consider the effects and impact on caregivers.

The cost of each additional chronic condition increases both annual healthcare and social care costs by SGD$2,265 and SGD$3,177 respectively. An increase in cost with increasing number of chronic conditions has also been observed in several other studies [3, 5]. Lehnert and colleagues [7] conducted a systematic review which investigated the relationship between multiple chronic conditions and healthcare utilisation and costs and found that total healthcare expenditures rose virtually exponentially with the number of chronic conditions, which further highlights the impact of multimorbidity on the healthcare system and society at large.

Higher total societal and healthcare costs were significantly associated with age. Those aged 75 years and above, would be expected to experience a 79 % and 48 % increase in societal and healthcare costs respectively, while those aged 85 years and older would see a 427 % and 261 % increase in societal and healthcare costs, highlighting that age is a strong predictor of overall costs. Whilst we did not observe an impact on gender, this and age are commonly found to affect differences in healthcare utilisation and costs between those who are and are not multimorbid [3, 20, 37].

Indian ethnicity was also found to be associated with significant increases to societal care costs. Whilst it is difficult to elucidate these findings in terms of increased costs from just the societal perspective and not healthcare costs, it may be explained by certain ethnic and cultural differences. Given that societal costs are inclusive of both costs to the healthcare system and costs to society, we speculate that these differences are driven by informal care provided via help with ADLs and IADLS. It is possible that involvement of informal care providers differed across the different ethnic groups leading to the observed differences. Being retired was predicted to increase societal costs by 139 % compared to those who were employed. Given that being retired was also significantly associated with multimorbidity and multimorbidity incurred significantly higher costs, this is likely to explain the increased costs to society.

Our study findings should be viewed in light of the following limitations. This study relied on self-reported chronic conditions and service utilisation and the true prevalence of multimorbidity and the associated healthcare utilisation and cost may therefore be under or over reported. We only included a list of 10 chronic conditions in our study and therefore if additional chronic conditions were included, the prevalence and impact of multimorbidity would be expected to be even greater. We were not able to include costs of health insurance (e.g., any plan, Medishield, Eldershield) as associated variables were largely incomplete. As there are no published local unit costs for Singapore, the use of international estimates needed to be adopted in order to derive Singapore costs. However, this practice is a widely accepted for performing economic cost estimations [38]. We also did not consider the impact of specific disease combinations or the severity of specific chronic conditions in relation to healthcare utilisation or costs. Finally we acknowledge that the prediction model values are relatively low, however given the focus of the study was not on a prediction model, but rather the relationship between dependent and independent variables to identify the major contributors to the total cost, the findings are still valid and of great importance.

## Conclusions

This is the first study in Singapore to look at multimorbidity and associated healthcare utilisation and societal costs among older adults and one of only a few to look at costs from the societal perspective. Additional strengths of our study include the large sample size, inclusion of a representative sample of the general population, good response rate, use of widely accepted assessments and questionnaires that were cognitively tested, the option of completing the survey in numerous languages and dialects and the superior quality control measures and processes that were implemented throughout the study duration.

Given that more than half the older adult population in Singapore has two or more chronic conditions, the issue of multimorbidity needs to be addressed. For every additional condition, this is likely to be compounded with additional healthcare appointments, medications, as well as impact on various aspects of social care. The coordination of care of such people poses increased challenges and strategies to better manage such cases are required. As multimorbidity is becoming the norm more so than the exception, particularly with an ageing population, we need healthcare systems that can address these emerging challenges associated with multimorbidity and the respective healthcare and societal costs. While individual diseases dominate healthcare delivery, those with multiple chronic conditions need a broader approach. A better understanding of the epidemiology and complexities of multimorbidity is necessary in order to develop interventions to prevent multimorbidity, reduce its burden, and align healthcare services more closely with patients’ needs [4].

A rapidly ageing population has implications on the old-age support ratio, which has steadily decreased from 13.5 working adults to every person over 65 years in 1970 to just 5.7 in 2015 [39], resulting in fewer informal and professional caregivers as well as health care professionals being able to provide care for older adults, placing added pressure on existing care providers. In addition, over time we have seen increased participation of women (who more traditionally provided the bulk of unpaid care) in the paid workforce and the trend towards formation of nuclear families, both which have public policy implications, as such movements will impact the capacity to provide supportive care. Consequently, there will be a growing demand for more costly formal care arrangements to support an ageing population and their caregivers and therefore the current healthcare system needs to be prepared and sufficient to deal with these added pressures. In order to address the challenges and constraints of an ageing population, efforts need to be made to improve preventative healthcare. At the same time, novel needs-based medical health and long-term care models should find a balance between the goals of the patient, caregivers and family, healthcare providers, and the healthcare system [40].

### Availability of data and materials

Data is not available for online access, however readers who wish to gain access to the data can write to the senior author Dr Mythily Subramaniam at mythily@imh.com.sg with their requests. Access can be granted subject to the Institutional Review Board (IRB) and the research collaborative agreement guidelines. This is a requirement mandated for this research study by our IRB and funders.
